# Supplementary material for: Identification of photoperiod-induced specific miRNAs in the adrenal glands of Sunite sheep (Ovis aries)
Source: Front Vet Sci. 2022 Jul 22;9:888207. doi: 10.3389/fvets.2022.888207 (PMC9354845; doi:10.3389/fvets.2022.888207)
Supplement: Supplementary file 1 [file Data_Sheet_1.ZIP › Supplementary file/miRDeep2 file.zip!/miRDeep2 file/Novel_Structure/LP42A3/Novel_279.pdf]

| | |
|---|---|
| Provisional ID | : NC_019470.2_15057 |
| Score total | : 26.5 |
| Score for star read(s) | : 3.9 |
| Score for read counts | : 19.5 |
| Score for mfe | : 1.5 |
| Score for randfold | : 1.6 |
| Score for cons. seed | : |
| Total read count | : 50 |
| Mature read count | : 46 |
| Loop read count | : 0 |
| Star read count | : 4 |

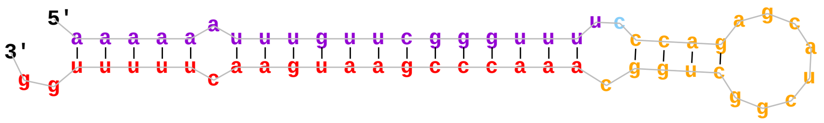


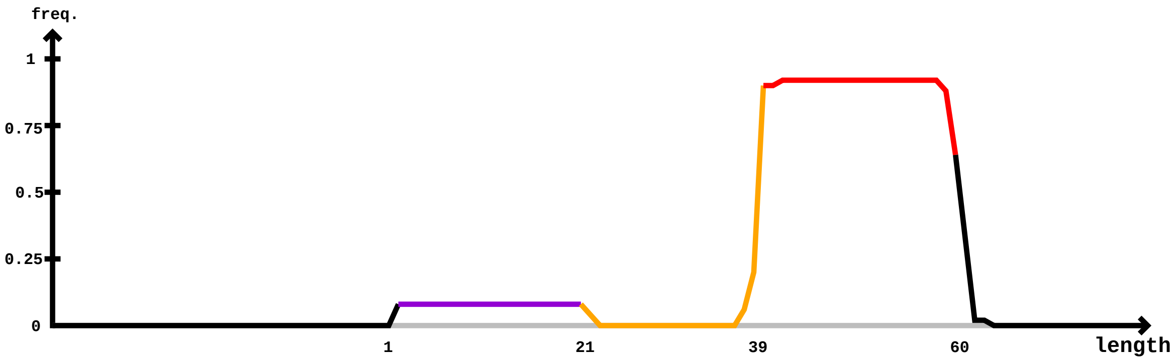


**Star** **Mature**

```
5'- guguugaucuaacacacuauaucucagguuggucaaaaaauuuguucggguuuucccagagcaucggcuggcaaacccgaaugaacuuuuuggucaacucacuacucuggca   -3'    obs
    guguugaucuaacacacuauaucucagguuggucaaaaaauuuguucggguuuucccagagcaucggcuggcaaacccgaaugaacuuuuuggucaacucacuacucuggca          exp
    ((((((...))))))...........(((((((((((((.(((((((((((((..((((........)))).))))))))))))).)))))))))))))).............    reads       mm          sample
    ..................................aaaaaGuuuguucggguuuu..........................................................    4           1           seq
    ......................................................................gAaaacccgaaugaacuuuuu.....................    1           1           seq
    ......................................................................gcaaacccgaaugaacuuuuug....................    2           0           seq
    .......................................................................Aaaacccgaaugaacuuuuug....................    6           1           seq
    .......................................................................caaaccUgaaugaacuuuuugg...................    1           1           seq
    ........................................................................aaaccAgaaugaacuuuuu.....................    1           1           seq
    ........................................................................aaacccgaaugaacuuuuuA....................    3           1           seq
    ........................................................................aaacccgaaugaacuuuuuC....................    1           1           seq
    ........................................................................aaacccgaaugaacuuuuugg...................    23          0           seq
    ........................................................................aaacccAaaugaacuuuuuggu..................    1           1           seq
    ........................................................................aaacccgaaugaacuuuuuggu..................    6           0           seq
    ..........................................................................aUccgaaugaacuuuuugguca................    1           1           seq
```
